# (Re)production of Inequalities in Retirement Practices and Meanings Assigned to the Term ‘Retiree’ in the Post-Communist Context

**DOI:** 10.3389/fsoc.2021.686674

**Published:** 2021-06-28

**Authors:** Anna Urbaniak

**Affiliations:** Institute of Sociology, University of Vienna, Vienna, Austria

**Keywords:** transitions, retirement, inequalites, practice theories, qualitative research, stagnant retiree, new wave retiree, post-communist

## Abstract

In the institutionalized life course transition from work to retirement is the transition that culturally defines the beginning of later life. However, there is no universal way of experiencing retirement or understanding retirees’ social roles. Especially in the context of the post-communist, liquid modern reality in Poland. The social role of the retiree, defined as a set of rules and expectations generated for individuals occupying particular positions in the social structure, is constructed at the intersection of what is culturally defined and individually negotiated. Therefore, the way in which individuals (re)define term “retiree” and “do retirement” reflects not only inequalities in individual resources and attitudes, but also in social structure in a given place and at a given time. In this contribution, I draw upon data from 68 qualitative interviews with retirees from Poland to analyze retirement practices and meanings assigned to the term “retiree.” Applying practice theory, I explore the inequalities they (re)produce, mirror and reinforce at the same time. Results show that there are four broad types of retirement practices: caregiving, working, exploring and disengaging. During analysis of meanings assigned by participants to the term “retiree,” two definitions emerged: one of a “new wave retiree” and the other of a “stagnant retiree.” Results suggest that in the post-communist context, retirement practices and meanings assigned to the term “retiree” are in the ongoing process of (re)negotiation and are influenced on the one hand by the activation demands resulting from discourses of active and productive aging, and on the other by habitus and imaginaries of retirement formed in the bygone communist era. Retirement practices and definitions of the term “retiree” that emerged from the data reflect structural and individual inequalities, highlighting intersection of gender, age and socioeconomic status in the (re)production of inequalities in retirement transition in the post-communist context.

## Introduction

The transition from work to retirement might serve as a risk point for older adults by multiplying individual and structural inequalities and/or creating new ones (Dewilde, 2012; Grenier, 2012). Major transitions in older people’s lives can lead to developing new social roles assigned to statuses acquired as result of transitioning processes. Literature refers to this process of getting adjusted to a new status as the incorporation stage of the transition (Van Gennep, 1960). Transitioning into retirement might involve ending a life-long identity built around the working role (Bordia et al., 2020) and is considered as one of the cultural markers that indicates beginning of old age (Irwin, 1999). As such, it poses lots of questions around inequalities concerning roles and practices developed in incorporation stage of retirement transition and transitional outcomes in different life spheres (Bosworth et al., 2016; Fasang, 2012). I argue that the (re)distribution of these inequalities is incorporated into the meanings and (re)negotiations of retirees’ roles and retirement practices^1^. Following the practice theory approach, I trace inequalities in practical understandings (Schatzki, 2002) of “doing retirement” and defining term “retiree” in a post-communist context.

In the 1960s, retirement was considered a time of stepping down and disengagement that starting from professional roles and stretching out beyond, also encompassing other areas of life (Cumming and Henry, 1961). Therefore, retired, older people were referred to as “roleless” (Burgess, 1960). This understanding of retirement as a period of limited activities and withdrawal from social roles was also common in Poland during the communist regime (Jałowiecki, 1973; Czerniawska, 1998). Now, as the population is aging, the meaning of older age and retirement is changing. Public discourse on active aging (Walker and Maltby, 2012; Boudiny, 2013) and policies of extending working life (OECD, 2006; Berkman et al., 2015), highlight that the concept of older people as “roleless” is far from the truth (Vidovićová, 2018). Changes around retirement practices and meanings assigned to the term “retiree” are influenced by individual and structural factors. I argue that with profound changes in the welfare system (Śleszyński, 2017), Poland is an interesting case study of sociocultural inequalities and tensions around retirement in post-communist regimes (Vanhuysse, 2004; Libman & Obydenkova, 2019), as individual transitioning into retirement was entangled with structural transitioning into a post–communist regime and/or its long-term consequences.

So far there has been little consideration of how retirees experience retirement transition in a liquid post–communist context (Krzyżowski, 2011; Krzyżowski et al., 2014). Especially the analysis of inequalities (re)produced in retirement practices and meanings assigned to the term ‘retiree’ was missing. This paper reports on an exploratory study aimed at addressing these deficits. It examines inequalities in practices of ‘doing retirement’ and (re)negotiating meanings assigned to the term “retiree.” Unpacking these inequalities is important to better understand retirement transition in a post-communist context.

In this contribution, I draw upon data from 68 qualitative interviews with retirees from Poland to analyze retirement practices and meanings assigned to the term “retiree.” Applying practice theory, I explore inequalities embedded in the practical knowledge (Schatzki, 2002; Kustermans, 2016) of doing retirement. In doing so, I analyze retirement practices and meaning retirees assign to the term “retiree.” I argue that this allows one to capture how the roles of retirees are (re)constructed at the intersection of what is culturally defined and individually negotiated in the post–communist context.

The paper is structured into four parts: the first part outlines theoretical insights on retirement as a life transition and the contexts of transitioning into retirement in a post-communist jurisdiction. The second part introduces the study “Early Stage Retirees in Urban Environments in Poland” and the data analyzed in this contribution. The third part presents the results, and the fourth part discusses them.

### Retiring as a Major Life Transition

In this contribution, retirement is understood as a transitional process that occurs within the individual’s life course and incorporates objective transformation as well as subjective developmental change in identity, expectations, preferences, and meanings. Retiring as a transition involves changes in the material situation, social status, social networks, social roles, identity, and lifestyle (Mortimer and Moen, 2016). Whereas those changes might have positive as well as negative outcomes for individuals, there is a longstanding recognition of the importance of the major life transitions, such as retirement, on (re)production of inequalities. Current literature highlights that individual and structural forms of (dis)advantage are accumulated during the lifecourse and transitions can lead to further inequalities in old age (Dannefer, 2003; Dewilde, 2003; Scharf et al., 2005). It’s also argued that (dis)advantage experienced across the lifecourse can impact subjective interpretations of transitions in later life (Grenier, 2012).

There is a growing international body of evidence on retirement impacts on inequalities across multiple domains of life such as: services, amenities and mobility, (e.g. Peace & Holland, 2001), material and financial resources, (e.g. Tchernina and Tchernin, 2002), sociocultural aspects, (e.g. Laliberte Rudman, 2015), civic participation, (e.g. Ní Léime et al., 2015), and social relations, (e.g. Patulny, 2009). There are also numerous publications testifying to the role of individual resources having an impact on individual experiences of retirement. According to a recent review of factors contributing to retirement adjustment by Barbosa et al., 2016, the most researched dimensions contributing to different experiences of retirement transitioning and their outcomes for individuals are: physical health, finances, psychological health and personality-related attributes. Their review also highlights the roles of leisure, voluntary retirement, and social integration in the retirement transition experience. The authors conclude that to extend the knowledge on retirement transitions, it is necessary to investigate further under-researched domains of institutional, social, and cultural elements, and among others, the perception of retirees’ roles.

The perception and conceptualization of the “retiree” has changed across time, from being viewed as the loss of a social roles (Burgess, 1960), through the continuity of roles (Atchley, 1989; Quick and Moen, 1998), and currently it’s recognized as a period of possibilities/pressures to acquire multiple new roles (Vidovićová, 2018). Within the discourse of productive aging, activity in older age is considered beneficial (Taylor and Earl, 2016), which contributes to the development of activation demands that new generations of retirees are facing and need to respond to (Tomasik and Silbereisen, 2014). As a result, with time, along with the population aging, the transition into retirement has become “fuzzier” (Kohli et al., 1991) and (re)produces numerous inequalities. There is a longstanding tradition of understanding retirement as a transition that involves the evolution of social roles, (e.g. Crawford, 1973; van den Bogaard, 2017). There is also a recognition that social roles are important for people transitioning into retirement (Moen et al., 2000; Heaven et al., 2013; Phyllis) as they contribute to well-being of individuals and impact wider communities (Musick and Wilson, 2003; Lum and Lightfoot, 2005). Despite that, however, only a limited literature exists specifically addressing the way in which individuals (re)define the term “retiree” and experience retirement in the post-communist context.

Krzyżowski (2011), analyzing the influence of social roles on retiring strategies, notes the role of cultural patterns and their individual interpretations. He highlights that individual interpretations of retirees’ roles are gendered and rooted in their sociocultural context. Krzyżanowska (2011) illustrates the mixed influence of understandings of old age and leaving the labor market on attitudes toward retirement. In their conceptual framework of old-age culture in Poland, Krzyżowski et al., 2014 highlight the role of individual resources in how inequalities in retirement are distributed and concluded that these inequalities determine the level of activity in old age. All of the publications mentioned analyze the meanings and (re)negotiations of retirees’ roles in the post-communist context follow to some degree lines drawn by the most often applied theoretical perspectives of retirement as a form of decision-making and/or retirement as an adjustment process (Wang and Shultz, 2010).

Aiming to contribute to the body of evidence on experiencing retirement in post-communist contexts and expand inquiry beyond limitations incorporated into existing theories, this contribution follows the recent call to explore the potential of practice theory in advancing our understanding of the retirement transition (Wanka, 2019). In the line of argumentation offered by practice theories, the practice of retiring and retirement practices are not just done by the retiree alone; the material, institutional and cultural settings in which the retiring and being retired takes place and the idea of what it means to be a retiree (and motivations for it) are all equally involved in creating the practice of retiring and retirement practices. In a practice-theoretical framework, retirees are produced through practices: “They understand the world and themselves and use know-how and motivational knowledge according to the particular practice” (Reckwitz, 2002, p. 256). In this contribution, I focus on the incorporation stage (Van Gennep, 1960) of the retirement transition and argue that to advance empirical analysis of inequalities in the retirement transition means to reflect on certain practices that create the experience of retirement and on understanding of the term “retiree.”

The context in which retirement is “being done” and the practical knowledge of being a retiree is being constructed is framed to some degree by policy regulations, (e.g. statutory retirement age, available pathways to retirement) and public discourse (around retirement, retiree roles and aging as such). A growing body of evidence highlights how discourses framing active and productive aging are highly discriminatory with regard to income, education, health status, and gender (Marshall and Katz, 2016; Calasanti and Repetti, 2018). Kohli and Rein. (1991) analyzed the impacts of making the retirement transition in earlier life stages on well-being, and also considered the legitimacy of different institutional pathways and their potential for “stigmatisation,” highlighting the complex distribution of inequalities in retiring and being retired.

Therefore, retirement, which is a turning point in an individual’s life, should be considered as a processual, practical accomplishment that involves various social practices, sites, and human, as well as non-human, actors (Wanka, 2019). This processual perspective of the socially constructed accomplishment entails inequalities from both individual and structural dimensions (Moen, 1996; Elder, 2003; Phyllis). The moment when individuals formally leave the labor market (practices of retiring) and the way they organize their lives during the incorporation stage of the transition (retirement practices) (re)construct inequalities from the perspective of individual biographies (Dingemans and Henkens, 2014) and also from the perspective of the functioning of wider entities as communities or societies. The context in which individuals make the transition to retirement has important implications for individual perceptions of what it means to be retired (Ekerdt, 2009, 2010). I argue that those inequalities are (re)constructed in definitions of the term “retiree” and in retirement practices. In turn, they both can be considered as yet another dimension of inequalities involved in the retirement transition. This is especially visible in the context of the post-communist regime in Poland, characterized by ever-changing pension system and competing imaginaries of retirement.

### The Liquid Reality of Retiring in Post-Communist Poland

Retirement practices and understandings of retirement can be perceived as being (re)negotiated and emerging at the intersection of individual resources and the structural setting. As previously mentioned, I consider Poland to be a very interesting site to analyze meanings assigned to the term “retiree” and retirement practices as both structural and individual circumstances remain in flux due to the consequences of the major socioeconomic transition from a communist to a democratic regime in 1989. In this context, individual life course transitions (retirement) have been entangled with deep structural changes resulting from a socioeconomic transformation (Trafiałek, 1998; Krzyżowski, 2011). This entanglement of individual and structural transitions magnifies the (re)production of inequalities in individuals’ experiences of the retirement transition. To unpack individual retirement experiences, presented in the next part of this contribution, here I provide a brief overview of the structural setting of the Polish pension system.

Until 1998, there was a pay-as-you-go pension system with defined benefits in Poland. In pay-as-you-go schemes, contributions of working people finance the current pension payments. In the defined benefit system, the amount of one’s pension depended on the amount of earnings from selected years of their professional career and the period of insurance coverage. In this system, there were many opportunities to retire earlier. This meant that, despite the retirement age of 60 for women and 65 for men, the effective retirement age was about 5 years lower at the time (Zieleniecki, 2012). The social security system operating in Poland until 1998 was criticized as costly and unfair, and for being in danger of financial collapse (Góra and Rutkowski, 1997). The main disadvantage of the system was a number of incentives for early exit from the labor market and numerous pension privileges for various professional groups. Early retirement was often a way to avoid unemployment during the difficult period of transformation (Trafiałek, 1998) Therefore, the number of pensioners increased rapidly, which in turn created additional challenges to the stability of the pension system and the economic growth of the country (Chlon et al., 1999).

Consequently, the reform introduced in 1999 aimed mostly to ensure the financial stability of the system and maintain its solvency despite the high deficit of the system at the starting point and the gradual aging of the population. This required, on the one hand, the restoration of realistic proportions between the amount of the pension and the value of contributions paid by the insured person and, on the other hand, the creation of a system which would ensure that the inflow of these contributions would be significantly higher than under the system conditions before the reform. It was assumed that this will be possible by creating incentives in the pension system to increase labor activity and reduce the gray economy, as well as by creating a funded pillar of the system which will stimulate economic growth, thanks to the profits from investments in the financial market, and provide relatively higher pensions. The creators of the reform also believed that the introduction of two sources of financing, through risk diversification, would make it possible to increase the security of future payments (Góra and Rutkowski, 1997; Gomułka, 2014).

After this first change, the post-communist pension system was subject to numerous adjustments spread over time. Detailed analysis of the motives and consequences of these changes exceeds the scope of this study. To highlight how volatile the system was, Table 1 presents the brief overview of changes introduced in pension system between 1999 and 2017.

**TABLE 1:** Changes in pension system in Poland between 1999–2017.

Year	Scope	Description
1999	Social security system reform	This reform replaced the former system based exclusively on the “intergenerational solidarity principle” with a mixed system combining “defined benefit” and “defined contribution” arrangements. The new three-pillar system was principally defined by two mandatory pillars: “Mandatory public” was provided by the social security institution (zakład ubezpieczeń społecznych, ZUS), and “mandatory private” by the open pension funds (otwarte fundusze emerytalne, OFE). While older citizens could choose whether to remain in ZUS or to share their contribution between the ZUS and OFE, those born in or after 1968 were obligated to join the two-pillar system. Finally, the reform created the third pillar (“voluntary private”), to be effectuated in relation to company pension plans
2000	Increase the attractiveness of employee pension programs	Due to the Act of March 2, 2000, amending the Act on employee pension programs and certain other acts, the financing of the basic contribution was changed; previously it was paid from the employee’s salary, but in 2000 it began to be financed by the employer
2003	Adjustments regarding benefits for uniform services	Uniform services were excluded from the general system and returned to the supply system
2004	Increase the attractiveness of voluntary savings	The Act of August 27, 2003 amended the Act on the organization and operation of pension funds, while other acts initiated a number of changes in provisions regarding fees, including the possibility of differentiation of fees due to the membership period being annulled or expired. Another change concerned the benefits collected by PTE on OFE assets. An act on individual retirement accounts also became applicable
2005	Adjustments regarding benefits for miners	Introduction of privileges for miners. They pay contributions within the general system; however, their pension is not linked to the contribution amount
2009	Introduction of bridging pensions	It allowed for paying a pension to people working in special conditions that end their professional activity before reaching the effective retirement age
2011	Reduction of contributions to OFE	Lowering the amount of premiums to OFE from 7.3 to 2.3%
2013	Increase of retirement age	Until 2013, the retirement age was defined separately for men and women at ages 65 and 60, respectively. From January 1, 2013, the retirement age was supposed to be increasing by a month in January, May and September each year until it reaches 67 for both sexes (women in 2040, men in 2020)
2014	Further changes in OFE	OFE were made to transfer 51.5% of their assets and all of their government bonds to ZUS, and they were forbidden from investing in government bonds in the future. Between April 1, 2014 and July 31, 2014, every person insured in the second pillar funds had to decide whether to keep their contributions there or to transfer all of them to ZUS; contributions remaining in the second pillar would be gradually transferred to ZUS in a process beginning ten years before the person’s retirement
2017	Changes in general pension system	Return to the constant retirement age at the age of 60 for women and 65 for men. Changes in the amounts of pensions: Raising the minimum pension amount and valorisations

Adopted from: A. Owczarczyk (2019), p. 150.

The stability of the existing pension system is still in question ((Bednarczyk and Raszewski, 2017), which might diminish individual agency and control in retirement transition, thereby reinforcing uncertainty in retirement decisions and contributing to ongoing processes of (re)negotiation and (re)definition of retirees’ roles, practices of retiring, and retirement practices.

In research on retirement transitions in Poland, in-depth studies on lived experiences remain limited (Kowalska, 2015; cf.; Krzyżowski et al., 2014). The existing body of evidence focuses primarily on the macroeconomic factors contributing to retirement decisions, possible pathways to retirement, and their outcomes for retiring individuals (Chybalski, 2013; Olejnik, 2016; Perek-Białas, 2017). Existing research tends to concentrate on retirement decisions and their context. Research show that employers often do not invest in older employees, limiting their opportunities to acquire competences needed in the ever-changing labor market (Perek-Białas et al., 2011; Stypińska, 2011). Consequently, they might be perceived as not equally valuable employees, which pushes them to retire. The research also shows that systematic solutions facilitating the gradual withdrawal from professional roles are limited and employees often remain unaware of such opportunities. Even though retirement is now perceived as a process that unfolds over time, rather than as a one-time discrete event with individuals transiting from full employment to full retirement and increasing proportion of workers engaging in bridging employment and unretirement (Fisher et al., 2016; Krejcova & Rašticová, 2020). Bridging employment, which is common in Western European countries, the United States, and Canada (Gobeski & Beehr, 2009; Von Bonsdorff et al., 2009) is not considered that often in Poland (Rzechowska, 2010). In 2013, only 8.7% of the population aged 50–69 declared a reduction in the number of working hours before deciding to retire (GUS, 2013:50). This all overlaps with the fact that the vast majority of retirees do not plan extensively for retirement and might experience this transition as a major rupture for which they were not fully prepared (Szatur-Jaworska et al., 2006). This refers also to economic aspects of becoming a retiree.^2^ As such, many do not know how to organize their own lives in this incorporation stage of the transition. Unpacking how individuals (re)define term “retiree” and “do” retirement in this liquid structural setting is critical to better understand the (re)production of inequalities related to the retirement transition in this post-communist context.

## Methods

Data presented here come from the mixed-methods project “Early Stage Retirees in Urban Environment in Poland: Social Contexts of Activity Patterns” (2011–2015) that analyses the social context of retirees’ activities in the urban environment in Poland. The project deployed qualitative and quantitative methods, gathering data on different activities of individuals who were in the incorporation stage of the retirement transition. The main focus was on understanding what activity means in the context of retirement (quantitative data analysis, desk research and public policy analysis) and how it is performed in the urban environment. In this contribution, only data from the qualitative component of the project are analyzed.

The qualitative component of the research consists of 68 individual in-depth interviews with people who lived in an urban environment (second-largest city in Poland) and received pension benefits. Those were the only predefined inclusion criteria for the sample, as the main focus of this qualitative part of research was on the choice of participants providing new insights and not on building a representative sample (Kaufmann, 2010). In order to theoretically saturate research categories, the choice of subsequent participants was dictated by their significance in relation to the data already gathered in completed interviews. For this reason, subsequent participants were selected on the basis of both similarity (a similar situation) and contrast (an extremely different situation) in relation to those characterized in the course of the conducted analysis (cf. Glaser and Strauss, 2017). This strategy allows for filling in the gaps and developing research intuitions that emerge during the analysis. Beyond the formal selection criteria, an equal gender balance, as well as heterogeneity in regard to marital and health status as well as material situation, educational attainment, former occupations, and pathways to retirement, (e.g. from unemployment, early retirement) were considered. As the experiences of retiring were diverse, the final sample includes people who retired at different stages of their life and those already retired at the time of interview from three months up to 10 years. There were 30 males and 38 females included. The youngest participant was 58 years old and the oldest 78 years old. Table 2 presents sociodemographic characteristics of the participants.

**TABLE 2:** Sociodemographic characteristics of the participants.

Age	Years of birth	1936–1955
Gender	Male	30
	Female	38
Primary occupation	Administrative and office staff, secretaries, post office workers, receptionists, telephone operators	7
	Creative and higher education professions, engineers, doctors, lawyers, teachers	22
	Directors, presidents, and managers of companies and institutions and state and local government administration	15
	Employees of shops, service points, personal services, security, conductors, babysitters, drivers	9
	Owners and co-owners of companies, plants, sales outlets, and other non-agricultural department managers	4
	Skilled workers and foremen employed outside agriculture and forestry	5
	Technicians and other associate professionals, nurses, non-commissioned officers, police officers	2
	Workers carrying out simple work outside agriculture and forestry, cleaners, caretakers, support workers	4
Marital status	Married/in partnership	43
	Single	6
	Divorced	4
	Widowed	15
Pathway to retirement	From full-time employment	12
	From part-time employment	11
	Early retirement	21
	From disability pension	2
	Continues to work while retired	17
	From unemployment	5

Participants took part in in-depth interviews that lasted approximately one and half hours and consisted of three parts: an open narrative biographical portion, a semi-structured portion and a map exercise where participants worked with the researcher to map out places and parts of the urban environment they choose for different types of activities. The maps were used to probe for differences in experiencing the urban environment during their retirement trajectory.

The author conducted 53 interviews with the support of research assistants, who carried out 15 interviews. All interviewers were experienced with qualitative interviewing and their background was in sociology. All interviewers were trained in using interview guides and the map exercise. The interview guide was tested in two pilot interviews that were not included in the final sample. Participants were primarily approached through gatekeepers or by interviewers in public spaces. Participants that were approached by gatekeepers were contacted by researcher via phone to establish the details of the time and place of the interview. Each participant was informed about the purpose of the research and what participation in the study involved. All participants expressed informed consent prior to the data collection and agreed to audio recording the interviews. None of the participants withdrew from the participation at any stage of the study.

Participants decided on the most convenient time and location of the interview. Researchers contacted participants via phone to confirm date and place of the interview one day before the scheduled meeting. Two participants wished to postpone the interviews due to unforeseen circumstances and participated in the interview at a later time. Interviews were conducted between March and November 2014 in Kraków. The majority of interviews (40) were conducted in researcher’s office at the university. 20 interviews were conducted in public spaces (parks, cafes, and restaurants) and eight at participants’ homes.

Interviews took place with only researcher and interviewee present, with nobody else present during the interview. In two cases, there were partners of interviewees present in the home, but not in the room where the interview took place. Field notes were taken during and after the interviews.

Collected data were transcribed by the researcher and research assistants. Data were anonymized and later analyzed by the researcher using MAXQDA software to code the data and derive themes from them. The following seven main themes emerged during the analysis: 1) retirement trajectories, 2) definitions and meanings assigned to the role of retiree, 3) definitions and meanings assigned to activities comprising “retiring” and “being retired,” 4) retirement as time for others, 5) retirement as time for oneself 6) the role of the urban environment in retirement transition, 7) challenges and opportunities faced by retirees in the urban environment.

For the purpose of this contribution, data were revisited to focus on retirement practices and meanings assigned to the term “retiree.” The main question leading this analysis was: what practices and meanings produce retirees in a post–communist jurisdiction? In the first step, following the practice theory approach, I analyze retirement through the lens of practices that construct retirement. In the second step, following the principles of semantic analysis, I reconstruct the definition of the term “retiree” emerging from the narratives. Based on the interviewees’ narratives, six separate networks for the keyword “retiree,” were created: 1) equivalents, 2) opposites, 3) terms, 4) associations, 5) actions by the subject and 6) actions toward the subject. The semantic field of the term “retiree” was created as a result of sorting out the separate networks within the field. The thematic or conceptual relationships and the functions of the phrases and actions used in the narratives made it possible to understand the fuller meaning of the concept of “retiree,” or the group of meanings in which it was used (cf. Dudkiewicz, 2006).

## Results

In this section, the main themes identified from interviews are presented. First, I explore retirement through the lens of “doing” by focusing on meanings assigned to four broad types of retirement practices: caregiving, working, exploring, and disengaging. This is followed by the definitions of term “retiree” reconstructed from participants’ narratives.

### Retirement as Caregiving

Retirees’ narratives highlighted the predominant presence of caregiving as a retirement practice in the post-communist context. The narratives clearly marked a different pattern of expectations and their implementation in the case of women and men. Women more often presented their retirement experience through the lens of caregiving practices than men did. There were 15 retirees providing care for older adults on the daily basis and 18 who provide care for their grandchildren. The retirees’ narratives have often reflected on caregiving for grandchildren as the most “natural” and deeply internalized retirement practice.

Aneta (born in 1949, retired in 2012), who started taking care of her grandchildren occasionally already when she was working part time, now provides care for her grandchildren on a regular basis. She highlights the emotional attachment she developed with her youngest grandson as well as her daughters’ expectations to provide care for him:

It (taking care of grandson) can be tiring, but I think […], it’s natural that my daughter expects me to help, you are expected to be granny I would put it that way [laughter]. And it’s a pleasure because, you know, you love your grandchildren (Aneta, at the time of interview was 65 years old, retired for two years).

Anastazja (born in 1947, retired in 2004) in her story illustrates even further the intersection of linked lives, gendered norms of care, and the wider economical context entangled with retirement practices in the post-communist context. Anastazja’s daughter wanted to develop her professional career in a highly competitive environment, which pushed Anastazja to retire earlier and take care of her granddaughter:

I practically raised Anna (granddaughter). Her mum needed to work a lot at that time so I stepped in. […] when she was little, she called me mum. (Anastazja, at the time of interview was 67 years old, retired for 10 years)

Among people experiencing retirement as caregiving, there were five people who provided daily care for both: older adult(s) and grandchild (ren). Bartosz (born in 1955, retired in 2008), who is the only man in this category, highlights the dynamic aspects of the sandwich generation’s retirement practices. Bartosz retired earlier to take care of his care-dependent mother after his father passed away. The additional task of taking care of his grandchildren was imposed on him and his wife in the fifth year of his retirement due to the sudden rupture in his daughter’s economic and family status. After the divorce, Bartosz’s daughter took up secondary employment and spends most of her time working in order to earn enough to be able to pay her mortgage:

There is no other way out, the daughter is left alone [after the divorce] with two children, and the wife and I have to take care of them (…), and they stay with us until their mum picks them up late in the evening (Bartosz, at the time of the interview was 59, retired for six years).

Joanna (born in 1952, retired in 2012), describing her retirement practices, focuses on caregiving for her 93-year-old godmother, who suffered a stroke. Since then, Joanna takes care of her daily and highlights how reciprocity impacts her caregiving practice:

I am looking after her (godmother) with all my heart (…). She has been good to me all my life and I want to pay her back as much as I can (Joanna, at the time of the interview was 62, retired for two years).

Lech (born in 1948, retired in 2012), who retired earlier due to heath issues, decided to provide care to his care-dependent neighbor, whose wife passed away. Lech reflects on the sense of moral obligation that motivates him, which is not rooted in personal reciprocity but in broader ethical terms:

I think this is an obligation to help an older person. I know if I don’t help him (the neighbor), nobody will. (Lech, at the time of interview was 66 years old, retired for two years).

### Retirement as Working

Whereas in the majority of narratives, retirement encompassed withdrawal from professional activities, in the narratives of 17 retirees (of whom 12 are men), the most important set of practices was related to experiencing retirement as working. The group of retirees working past retirement age can be split further into two groups: those with higher socioeconomic status, who worked in higher positions or were self-employed and those from a younger cohort with lower socioeconomic status. In the latter group, a majority of narratives framed working past retirement age as an economic necessity.

Eliza (born in 1950, retired in 2009), with a precarious working trajectory, highlights that in order to afford her everyday needs she requires more money than her pension benefits provide. Therefore, she continues employment past retirement age despite the desire to stop working:

I continue to work (…) in fact if it wasn’t for the money, I would not work. If the money were better, if only I had a higher pension, I couldn’t care less about working and this type of activity (Eliza, at the time of interview was 64, retired for five years).

In some cases, working past retirement age intersected with motivation to provide help to working family members highlighting another dimension of linked lives’ impacts on retirement practices. Marta (born in 1952, retired at 2010) agreed to work in her daughter’s grocery store, enabling her daughter to gain a skilled worker for a lower wage and at the same time allowed Marta to gain an extra income:

After I retired, because I had fairly low pension benefits, I worked for my daughter, I was helping her as I knew the job (…) (Marta, at the time of interview was 62, retired for four years).

As other narratives highlight, for those who worked in low-paid jobs before retirement, pension benefits are often a way of ensuring financial stability. Andrzej (born 1947, retired in 2012), whose working trajectory was interrupted by unemployment on numerous occasions, currently works as a support worker and reflecting on his situation, points out that for him retirement means working as usual:

I was immediately aiming for this, as soon as I got to retirement age I was simply going to retire and still work […] then my budget will improve significantly [compared to the situation] when I have just one [pension benefits or wage]. (Andrzej, at the time of interview was 67, retired for two years).

On the other hand, in the case of self-employed and people with higher socioeconomic status, working past retirement age was not perceived through the lens of economic necessity and often was experienced as an opportunity to maintain positive well-being and the identity of a productive member of the social system. Bogdan (born in 1942, retired in 2009), who continues to run family business while retired highlights the positive impacts of work on his well-being:

I always liked to work, and I still like it, and during periods when there is less work, I find myself falling into some sort of nostalgia, having too much time to think about negative things, so I prefer to have a lot of work so that I don’t focus on negatives. (Bogdan, at the time of interview was 72, retired for five years).

Some male participants, who perceive work past retirement age as a matter of choice, also highlight that working allows them to feel productive and keep their masculine identity of being doers. Zygmunt (born in 1948, retired in 2005), who works part-time in a managerial position in his son-in-law’s company, highlights that even if the work as such is not always pleasant, it provides him with the sense of meaning:

this work is neither easy nor pleasant, but it gives me an assurance that I feel I am still needed, that I’m still doing something. I'm not bored at home, I don't disturb my wife in the kitchen, I don't sit in a room or something like that (Zygmunt, at the time of interview was 66, retired for nine years).

### Retirement as Exploring

Narratives that focused on retirement as exploring were predominant among those who were: younger, at earlier stages of the retirement transition, and more often, women. Practices of exploring differ on many levels and incorporate diverse activities; however, they share the same focus on retirees’ needs to explore one’s own pleasures and to (re)discover oneself in the incorporation stage of the retirement transition.

Emilia (born in 1953, retired in 2013), who worked as an administrator, highlights that after getting involved in a church-led charity, volunteering became a way for her to (re)connect with her local community and share her skills beyond her previous work role:

Apparently, I must have some mission [laughter] and some purpose beyond my work. That is why I signed up for this project, to deepen my optimism, which I can pass on to others. I wanted to do something I have never done before […] it [project she volunteers for] is mainly addressed to the unemployed, to a hundred people from the area. (Emilia, 61 years old, retired for a year).

For others, as highlighted by Wacław (born in 1951, retired in 2009), who worked in an office, exploring meant to be more interested and proactive in discovering the cultural life that the city has to offer:

*Before retirement, there wasn't that much time, you had to earn a living (…). And there was always more work to do, because you had to earn some money. And now I have more time to go out whenever and wherever I want to. For example, to a museum or other interesting places. I just look for what is there. If I find something interesting, then I decide with my wife what will we do.* (Wacław, at time of interview 63, retired for five years).

The stories of Emilia and Wacław highlight that retirement as exploring might be experienced through activities that retirees were not able to be involved in while working. Most often, this was due to the fact that they focused primarily on their professional and/or family roles. Those two stories also highlight a more general difference between genders: whereas women were more likely to engage in organized activities and activities in the public space (participation in lectures at universities of third age, fitness classes, etc.), men more often presented their exploring through individual activities, sometimes limited to the privacy of their homes. Regardless of gender, retirees experiencing retirement as exploring highlighted the role of being selective and focusing on activities they considered interesting and valuable, as illustrated by Aleksandra (born in 1953, retired in 2008):

[…] I was going to the regional library, where for the whole time of summer holidays there were various courses every week. There was also a language course, but I wasn't interested in that, but for example, there was a computer course, a short two-day course then three days break, and another course I was interested in (Aleksandra, at the time of the interview 61, retired for six years).

For those who experience retirement as exploring, it was more common to take an active stand against age discrimination. For example, Tadeusz (born in 1950, retired in 2005) describes how he avoids the label of “retiree-friendly activities” while exploring his opportunities:

[…] but I am not particularly interested in what is for retirees, but in what is for people (Tadeusz, at the time of interview 64, retired for nine years).

For most retirees, experiencing retirement as exploring these practices translated into higher level of internalization of activation demands. Wiktor (born in 1947, retired in 2009) highlights how his long-standing interest in taking walks evolved into more conscious way of experiencing his environment motivated by an ambition to pass on knowledge on his neighborhood:

I started to take an interest in the way I went on those walks, because I started to take a detailed interest in a given monument I passed on my way or other elements, in order to have knowledge, to be able to somehow … pass that knowledge on to friends who came to visit us (Wiktor, at time of interview 67, retired for five years).

### Retirement as Disengaging

For some of interviewees, retirement was predominantly experienced through the practices of spatial and affective disengagement. In such stories, being retired was framed as being able to “do nothing” and “go nowhere” in contrast to the pre-retirement stage of life, filled predominantly with the activities related to the labor market and outside the home. In those narratives, retirees were opposed to activation demands and were inclined to rely on imaginaries of retirement shaped by the habitus of the bygone communist era. Łukasz (born in 1943, retired in 2008), who worked as a mechanic, refers to retirement as being an award deserved after years of work:

I will tell you, after so many years of work, after all this, I think I deserve a period of doing nothing, don’t I? […] (Łukasz, at time of interview 71, retired for 6 years).

Leaving professional obligations due to retirement in the experience of retirees creates the possibility to use time unproductively and some enjoyed this opportunity, which was not available during their working lives. For retirees who were more advanced in their retirement trajectories, the practices of disengaging and disengaging were framed as being able to rest, as Katarzyna (born 1938, retired in 2005) highlights:

I’m kind of lazy now because I am at ease, there is no longer any obligation to do so […] If I want to do something, I will do something, if not then I am resting, just like that. Let it be the whole day even. (Katarzyna, 76 years old, retired for 9 years).

Similarly, Artur (born in 1936, retired in 2004), who worked as a teacher and complained about the burnout related to being overworked, when reflecting on what being retired means to him highlights the positive well-being following disengagement:

I simply can stay home and do nothing, and I don't need anything more to be happy (Artur, 78 years old, retired for 10 years).

Both Katarzyna and Artur represent older cohort of retirees, whose expectations around retirement were influenced by the rules of old pension system and imaginaries of retirement developed in communist era.

For some of the interviewees, practices of disengagement were encompassed by their individual mindsets and/or health status. In this group, the macrostructural factors were often presented as important mediators of retirement practices.

Paweł (born in 1936, retired in 2011) before retirement worked part-time as a technician and highlights how his retirement experience is framed by his lack of interest in spending his time actively. He considers also the limited pension benefits that contribute to his withdrawal by limiting his possibilities:

When you get to this age, you will see … I don’t know what gets into a person, but you just don’t want to. You don’t want to do this, you don’t want to do that. You just don’t want to. And the government gives you nothing so you can’t afford much (Paweł, at time of interview 78, retired for 3 years).

Nina (born 1942, retired 2004), who worked in a store, reflects on her ill health, changes in the social network resulting from intersection of retirement and bereavement, as well as wider socioeconomic circumstances contributing to her disengagement:

A person cannot go anywhere [without a husband], there is no such thing as social life for me now as I’m retired and alone. […] it’s just such times that people somehow can’t afford to go out like they once did (Nina, at time of interview 72, retired for 10 years).

Narratives of some interviewees, who experienced retirement as disengagement, highlighted how important it was for them to ground their sense of entitlement to this passive way of spending time in a social contract framing older people as those who need to withdraw at least from the labor market in order to let younger people be fully engaged. Tomasz (born 1948, retired 2008) highlights that his disengagement results from perception of the need to “make space” in the labor market for younger generations:

[…] my wife who is younger is still working, and I’m already languid in retirement to give the space for the new generations, right? (Tomasz, at the time of interview 68, retired for six years).

### Reconstruction of the Definitions of the Term “Retiree”

Unpacking the practical knowledge underlying presented retirement practices requires further analysis of definitions and meanings assigned to retirees’ roles. Therefore, in these collected narratives, a semantic analysis of the term “retiree” was deployed. Each interview was analyzed according to Robin, 1980 guidelines. Table 3 presents findings of this analysis.

**TABLE 3:** Semantic field analysis of term retiree in collected narratives.

Type of retiree^a^	Equivalents Synonimy	Opposites	Terms	Associations	Actions by the subject	Actions toward the subject
Stagnant	- A Person of advanced age	- People in managerial positions	-is homebound	-Sofa	-requires help	-Employing as caregiver for grandchildren
	-An old person	-People who are influential	-is alone	-Slippers	-visits doctors frequently	-considering a second-class citizen
	- A sluggard		-has a period of professional prosperity behind them	-TV	-do not keep up with the world	- Being ignored by the government
			-is more likely a man	-Soap operas	-is unable to adapt to change, -lives in slow motion, -lags behind, -suffers from having too much free time	
				-Radio Maryja		
New wave	-A person who experiences second youth	-Grandparents	-is unconstrained by work	-Discounted prices of tickets	-wants to live	-Taking their advices
	-A person who has a green ID	-People who are working and have no life	- is more likely a woman	-Nordic walking	-has their own life	-Showing on television
		- home-bound people who are staring out on the streets	-is creative	-Swimming	-knows how to take care of one's health	-Liking
		-“Traditional” retirees, planting their roots in a sofa and wailing over their fate	-is intense	-Internet	-takes trips with friends, -keeps one's mind active, -works past retirement age	
		-Grandmothers with crutches	-is modern, is committed, is wiser, better prepared for life,	-Press and books	-has no time	
		-Grandparents in slippers	is younger in spirit			
		-The old	-is active			
		-The sick and the very disabled	-is at a higher level of development, is at peak			
		-Retirees with difficult life experiences	- is more powerful and self-sufficient, -is rather efficient			
		-Retirees living in the countryside	- is constantly busy with something			
			- is engaged in physical activity			

a Definitions were reconstructed on the basis of narratives but names were coined by the Author.

It became clear that interviewees in their narratives assign meanings to the term “retiree” in two sub-groups. Therefore, to capture this split, two definitions were reconstructed, as recognized by the interviewees^3^, who distinguished two types of retiree. Those opposite definitions capture opinions and experiences of interviewees and can be considered two ideal types that reflect inequalities in practical understandings of being retiree.

A stagnant retiree is a person of advanced age, a sluggard, an introvert, a person who suffers from having too much free time, somebody who is homebound, who is alone, who has a period of professional prosperity behind them, who is more likely a man, as opposed to people in managerial positions, people who are influential. The stagnant retiree complains, sits on a sofa, wears slippers, watches TV, watches soap operas, listens to Radio Maryja^4^. Being a stagnant retiree is associated with visiting doctors frequently, being terribly tied up with grandchildren, requiring help, not keeping up with the world and being unable to adapt to change, living in slow motion, lagging behind. The stagnant retiree is employed as caregiver for grandchildren, is being treated as a second-class citizen, as a burden and is not thought of by the authorities.

A new-wave retiree is a person who experiences second youth, who is unconstrained by work, who wants to live, who has a green ID^5^, benefits from discounted prices of tickets, who is more likely a woman, as opposed to the “grandparents” who take care of their grandchildren, people who are working and who do not live life, home-bound staring out on the street, “traditional” retirees, planting their roots in a sofa and wailing over their fate, grandmothers with crutches and grandparents in slippers, the old, the sick and the very disabled, old-type retirees with difficult life experiences, retirees living in the countryside. The new wave retiree is creative and intense, modern, committed, wiser, better prepared for life, younger in spirit, active, liked, at a higher level of development, at their peak, more powerful and self-sufficient, rather efficient. Being a new wave retiree is associated with having their own life, doing Nordic walking or swimming, knowing how to take care of one’s health, using the Internet, reading the press and books, taking trips with friends, keeping one’s mind active, working past retirement age, having no time. The new wave retiree is constantly busy with something, is engaged in physical activity, having their advice taken by others and being shown on television.

Presented definitions capture contrasting activities, different sets of skills, wishes and capitals between different types of retirees, reflecting inequalities (re)constructed in meanings assigned to retiree in the post-communist context. The fact that it was impossible to present one coherent definition of term retiree highlights that the understanding of retirees’ roles is still under construction and as the reconstructed definitions show, is influenced by imaginaries of retirement that have been developed in the context of activation demands on the one hand, and bygone communist era on the other.

The differences in the oppositions assigned to the term “retiree” across both definitions highlight that the demarcation line separates stagnant retirees from influential people in managerial positions and the new-wave retirees from the generation of what they labeled as “traditional” retirees. Those “traditional retirees” represent here retirees from imaginaries of retirement constructed in communist era when retirement was perceived as withdrawal and role of retiree was perceived as a “roleless” role.

## Discussion

This study sought to explore the way in which retired individuals (re)define term “retiree” and “do retirement” in a post-communist context. As argued in line with a practice-theoretical framework, subjects (retirees) are produced through practices (retirement practices). Therefore, the main focus of this contribution was to unpack the (re)production of inequalities in practices that frame the experiences of the incorporation stage of retirement trajectory and meanings assigned to the role of retiree in this post-communist context. Some authors, in analyzing the retirement experience, focus on a level of human agency that allows retirees to make choices that either adapt to, or react against, their context (Elder, 2003). Practice theories highlight that the agency is distributed across the network of employers, retirement schemes, colleagues, laws, families, bodies and health, and retirees themselves (Wanka, 2019). As I argued in this contribution, in the post-communist context, individual transitioning into retirement is entangled with structural transitioning and its long-term consequences. Therefore, it’s not so much the level of individual agency that constitutes the retirement experience in this liquid reality but rather the process of (re)inventing retirement through practices and meanings assigned to them.

The transition into being a retiree served as major turning point in most of participants’ lives and was experienced as a process involving sociocultural, institutional, and individual contexts and the interplay between them. This interplay impacted the distribution and (re)production of inequalities in experiencing retirement in this post-communist jurisdiction on many levels. While retirement practices were often diverse across individuals, in this contribution I focused only on four broad group of practices that emerged from participants’ narratives: caregiving, working, exploring, and disengaging. Below, I discuss findings on inequalities (re)constructed in these practices and meanings assigned to the term “retiree.”

Considered from a gender perspective, the retirement practices presented here revealed numerous inequalities. They were mirrored predominantly in narratives around experiencing retirement as working and caregiving. Interviewees’ narratives highlighted gendered inequalities in terms of external pressures and expectations (Titkow, 2004) as well as in terms of individual experiences of caregiving. The cases presented highlight how providing care for grandchildren was perceived mainly in terms of: economic necessity, a way to support one’s own children, an opportunity to build emotional relationships with grandchildren, or to transmit knowledge and skills. In providing care for older family members, interviewees most often highlighted the aspects of moral obligation in such practices. As the stories of Joanna and Lech demonstrate, practices of caretaking were not limited to immediate family members. Sociocultural expectations in post-communist countries associate the role of women with demands to provide care for children, grandchildren, and aging parents (Naldini et al., 2016). These expectations were strongly internalized by the women interviewed.

Whereas men in this study referred to their caregiving relationships in terms of providing practical support, women emphasized their emotional bond with grandchildren and the older adults they cared for. On the other hand, narratives of the twelve men in this sample presented working as central to their masculinity. This mirrors results suggesting that men reinforce their gendered identity as “doers” and providers (Ribeiro et al., 2007), and adds to the argument on maintaining such identity through retirement practices of working and internalization of activation demands. This study reflects the findings of previous work that has documented inequalities emerging at intersection of individual, sociocultural, and formal contexts testifying that women are more likely than men to forgo paid employment at various stages in the life course to be involved in caregiving roles (P. Moen et al., 1994; M. E. Szinovacz et al., 2001; Van Houtven et al., 2013). In the sample, the majority of women who experienced retirement as working continued to work as their retirement benefits were insufficient for their needs. This contributes to the body of evidence highlighting that involvement in caregiving roles leads to career and financial disadvantage (Presser, 2003) and contributes to greater inequalities between men and women (Hoque and Kirkpatrick, 2003). This also highlights the intersectional character of inequalities reflected in retirement practices.

Narratives gathered in this study testify that it is necessary to place the understanding of retirement practices in the wider context of previous life experiences (Elder, 1995) and existing networks (Elder, 2003). Following the idea of “linked lives” (Settersten, 2015) research, evidence shows that retirement decisions are not made in a vacuum but are influenced by the social context and significant others (Grenier, 2012; Eismann et al., 2019). Researchers highlight that especially the family context in which labor-market decisions are made is important for unpacking our understanding of the retirement transition (Denaeghel et al., 2011). Results presented in this study bring this argument further, showing that this impacts not only retirement decisions but also the retirement practices in the incorporation stage of the retirement transition. This contributes to the body of evidence highlighting that the degree to which lives are linked either through marriage, social networks, or family relations can influence an individual’s experience of retirement (Szinovacz and Deviney, 2000; Orrange, 2003; Schirle, 2008; Maximiliane E.). In the narratives analyzed, practices of caregiving and working were often linked with lives of significant others and were (re)constructed and (re)negotiated within social networks of retirees. Data show that in the liquid structural setting of the post-communist context, family structure might be (re)negotiated and roles (re)assigned depending on the individual and structural inequalities, as in case of Anastazja, who took over the role of mother for her granddaughter or Bartosz, who cares for his mother and grandchildren.

The practices presented here (re)construct inequalities in socioeconomic status that intersect with available resources, mediated by gender and social position. For participants with higher education, higher level of income, and in better health, retirement was often experienced as exploring practices that encompass active search for new roles, opportunities, developing new interests or deeper involvement in existing hobbies. For the majority of participants with lower socioeconomic status, exploring practices were not as established as their experience was mostly constructed by practices of working and caregiving. For participants with a precarious working trajectory, working practices were experienced as economic necessity. In the sample there were participants who retired at different stages of their life course, within different regulations of an ever-changing pension system. This lack of stability of the pension system created another source of inequality among people who were able to retire on given terms at a given time and those who were not. It multiplied inequalities resulting from gender, age, education, occupation and individual lifecycles.

The narratives also highlighted age cohort inequalities not only in terms of different possibilities created by the pension system but also in retirement practices. This was especially present in narratives around disengagement and attitudes toward activation demands. For those who were born earlier and whose social imaginaries of retirement were influenced by the communist heritage, retirement was predominantly constructed by practices of withdrawal and a low level of internalization of activation demands. As opposed to some earlier research showing that retirees might experience retirement as “anti-activity activities” (Katz, 2000) and frame them as a waste of time or guilty pleasures (Wanka, 2020), participants in this research often framed practices of disengagement as their entitlement and a desired way to experience retirement. I argue this is linked to habitus formed in the bygone communist era and reflects inequalities between expectations toward the retiree role created within the social imaginaries of different cohorts of retirees.

In debates around Polish culture of aging (Krzyżowski et al., 2014), national and local aging policies, (e.g. Błędowski, 2002), and in the offer of outlets available for retirees, (e.g. Pędziwiatr, 2015) two contrasting views on retirees’ role are presented. First, rooted in a concept of “role-less” role (Burgess, 1960) and the other incorporated in the vision of active and productive aging (Caro and Bass, 1993). To some degree, the definitions reconstructed from the narratives mirror this classification. The reconstructed definitions indicate that the meanings assigned to the role of retiree are ambiguous. Based on the clear division that interviewees drew between “new wave” and “stagnant” retiree, it’s possible to capture changes taking place in the area of retirement practices in post–communist contexts. This seems especially interesting when considering the distinctions marked in both definitions. Whereas in the definition of an “stagnant” retiree, the distinction is made on the basis of the opposition between a retiree and employee, in the case of the definition of a “new wave” retiree, it’s the opposition between two different types of retirees. This indicates that the reference point for defining retirees’ roles is shifting and captures the distinction between habitus shaped in the previous regime and habitus emerging now. The definition of the “new wave” retiree mirrors activation demands especially in the captured associations and listed actions of retirees that highlight physical and mental activity. Public discourse of active and productive aging seems to be captured by retirees in the definition of the “new-wave” retiree who is *being shown on TV.* Reconstructed definitions capture not only generational or age cohort differences but highlight also gendered inequalities, associating “stagnant” retirees rather with being a man and “new wave” retirees with being a woman. This result would require further investigation in other post-communist regimes.

The added value of looking closer at polish case in context of how retired individuals (re)define term “retiree” and “do retirement” in a fluid post-communist context emerges from the fact that Poland is the only one among OECD countries that has lowered its age of eligibility for retirement. In October 2017, Poland introduced new legislation to lower the official pension age to 65 for men and 60 for women. This is a significant policy reversal from the 2012 pension reforms which pro-posed increasing the official pension age to 67 years by 2020 for men and 2040 for women. It was a very specific, political decision, that plays a critical role in the reconstruction of the roles and images of retirement in Poland. This new political approach to retirement is rather unique among European countries as, contrary to models based on active aging policies, it favors rather disengagement. European Commission has raised concerns about these new reform, given that the decision goes against pension policy reforms in other EU countries that are increasing the pension age and moving toward gender equalization (Clemens and Parvani, 2017). As data presented in this study couldn’t capture impacts of this reform further research on how retired individuals (re)define term “retiree” and “do retirement” is needed that would focus specifically on inequalities related to gender and economical aspects of retiring. Comparative approach that would include more post-communist jurisdictions would be also needed in the future research.

As I was aiming to present in this contribution, analyzing differences in retirement practices allows for tracking the interplay of sociocultural, (i.e. gendered norms on caregiving) and institutional, (i.e. statutory retirement age) inequalities across individual lives. Findings from this study, although limited to one jurisdiction, highlight that some ways of understanding and going into retirement shaped in the communist context may be anachronistic considering the heterogeneity in retirees’ retirement practices and meanings assigned to them. Quotes from Łukasz and Tomasz, who while reflecting on their withdrawal practices tried to find reaffirmation of their views from the researcher highlight explicitly that retirement practices are inevitably (re)negotiated and (re)constructed in the reality that is intersubjective. Results presented here indicate that a once predictable pattern associated with aging and retirement is changing (Biggs 2005) and retirement transitions have become increasingly complex and new approaches are required to capture this complexity. Further unpacking how individuals (re)define the term “retiree” and “do” retirement in this liquid structural setting is critical to better understand inequalities related to retiring in a post-communist context. It is also critical in order to assess the capacity of existing institutional systems to support individuals during retirement transition.

## Data Availability

The datasets presented in this article are not readily available because dataset consists of in-depth interviews containing sensitive data of participants. Requests to access the datasets should be directed to anna.urbaniak@univie.ac.at.

## Footnotes

1 Retirement practices presented here should be distinguished from practices of retiring

2 In 2015 the average monthly pension in Poland was 2,170.64 PLN (ca 542 €) and the minimum of existence minimum at the level of 518.00 PLN (ca 130 €) (GUS, 2016: 35).

3 These definitions are based on the native language of the interviewees. Some of the culturally transmitted meanings might be impossible to translate into foreign language.

4 Radio Maryja is a religious and political socially conservative Polish radio station.

5 In Poland, those entitled to retirement receive an ID card that confirms their retiree status. This card is green in color.
